# False-negative BRAF V600E mutation results on fine-needle aspiration cytology of papillary thyroid carcinoma

**DOI:** 10.1186/s12957-017-1266-5

**Published:** 2017-11-13

**Authors:** Se Hyun Paek, Byung Seup Kim, Kyung Ho Kang, Hee Sung Kim

**Affiliations:** 1 Department of Surgery, College of Medicine, Ewha Womans University, Seoul, South Korea; 2 Department of Surgery, Hiu-Clinic, Suwon, South Korea; 3 Department of Surgery, Chung-Ang University Hospital and Chung-Ang University College of Medicine, 224-1, Heuk Seok-Dong, Dongjak-Ku, Seoul, 156-755 Republic of Korea; 4 Department of Pathology, Chung-Ang University Hospital and Chung-Ang University College of Medicine, Seoul, South Korea

**Keywords:** BRAF, False-negative rate, Fine-needle aspiration cytology, Papillary thyroid carcinoma

## Abstract

**Background:**

The BRAF V600E mutation is highly specific for papillary thyroid carcinoma (PTC). A test for this mutation can increase the diagnostic accuracy of fine-needle aspiration cytology (FNAC), but a considerably high false-negative rate for the BRAF V600E mutation on FNAC has been reported. In this study, we investigated the risk factors associated with false-negative BRAF V600E mutation results on FNAC.

**Methods:**

BRAF V600E mutation results of 221 PTC nodules between December 2011 and June 2013 were retrospectively reviewed. BRAF V600E mutation results on both preoperative FNAC and postoperative formalin-fixed, paraffin-embedded (FFPE) samples were compared. We investigated the sensitivity, specificity, positive predictive value (PPV), and negative predictive value (NPV) of BRAF V600E mutation results on FNAC. And, we identified the risk factors associated with false-negative results.

**Results:**

Of 221 PTC nodules, 150 (67.9%) on FNAC and 185 (83.7%) on FFPE samples were BRAF V600E mutation positive. The sensitivity, specificity, PPV, and NPV for BRAF V600E mutation testing with FNAC were 80.5, 97.2, 99.3, and 49.3%, respectively. Thirty-six (16.3%) BRAF V600E mutation-negative nodules on FNAC were mutation positive on FFPE sample analysis. Risk factors for these false-negative results were age, indeterminate FNAC results (nondiagnostic, atypia of undetermined significance (AUS), and findings suspicious for PTC), and PTC subtype.

**Conclusion:**

False-negative rate of BRAF mutation testing with FNAC for thyroid nodules is increased in cases of old age, indeterminate FNAC pathology results, and certain PTC subtypes. Therapeutic surgery can be considered for these cases. A well-designed prospective study with informed consent of patients will be essential for more informative results.

## Background

Thyroid nodules are the most common thyroid disease, with and incidence of approximately 1.5% in adolescents [1, 2]. Also, their incidence has been increasing probably due to more frequent use of thyroid ultrasonography as an initial thyroid nodule examination [3, 4].

Fine-needle aspiration cytology (FNAC) is currently the diagnostic tool of choice for evaluating thyroid nodules [5–7]. However, in as many as 30% of cases, FNAC-based evaluations of solitary thyroid nodules have a limited ability to discriminate between benign and malignant lesions; and results in an indeterminate cytological diagnosis [5, 8–10]. Consequently, planning optimal surgical management is challenging in patients with an uncertain preoperative diagnosis.

The BRAF V600E mutation is highly specific for papillary thyroid carcinoma (PTC) [11–14]. Several studies have demonstrated that BRAF mutation testing can increase the diagnostic accuracy of FNAC [12, 14–17]. The false-negative rate of PTC diagnosis can be reduced by routine BRAF mutation testing on FNAC specimens [14, 16]. The positive predictive value (PPV) of BRAF mutation testing is reported to be almost 100%; however, FNAC has been reported to have a considerably high false-negative rate for BRAF V600E mutation testing [14–17].

In this study, we investigated the risk factors associated with false-negative BRAF V600E mutation results on FNAC compared to corresponding formalin-fixed, paraffin-embedded (FFPE) surgical sample analysis.

## Methods

The BRAF V600E mutation results of 221 PTC nodules confirmed as PTC on permanent pathology between December 2011 and June 2013 were retrospectively reviewed. We performed BRAF V600E mutation testing on both preoperative FNAC and postoperative FFPE samples for all cases. The BRAF V600E mutation results on the preoperative FNAC sample were compared to those of the postoperative FFPE sample, and sensitivity, specificity, PPV, and negative predictive value (NPV) were determined. FNA slides with indeterminate results were re-reviewed by a cytopathologist blinded to the initial reading. Cases with discordant BRAF mutation test sites due to multifocal lesions were excluded (when there were several suspicious nodules before operation, BRAF testing was performed for the most suspicious nodule, and only FNA without BRAF testing was performed for the other nodules. Therefore, the number of nodules with preoperative BRAF mutation testing results will match the number of patients. In addition, only nodules with existing both preoperative and postoperative tests results were included.

Gender, age, mean tumor size, presence of extrathyroidal extension, lymph node metastasis, Bethesda classification, and PTC subtypes were evaluated and shown in Table 1. We investigated the risk factors associated with false-negative BRAF V600E mutation results on FNAC.

**Table 1:** Patients demographics

Variables	All groups (*n* = 221)
Gender (F:M)	179:42 (81.0:19.0%)
Age	46.6 ± 12.5 years
Tumor size	0.90 ± 0.70 cm
Extrathyroidal extension	73 (32.7%)
Lymph node metastasis	113 (50.7%)
Bethesda system	
Nondiagnostic	16 (7.2%)
Benign	0
AUS	11 (5.0%)
(Suspicious) FN	0
Suspicious	39 (17.6%)
Malignant	155 (70.1%)
Subtype of PTC	
Conventional	161 (72.9%)
Follicular variant	47 (21.3%)
Clear cell variant	3 (1.4%)
Oncocytic variant	7 (3.2%)
Tall cell variant	3 (1.4%)

All 221 PTC nodules that were subjected to FNAC (16 nondiagnostic, 11 atypia of undetermined significance [AUS], 39 suspicious PTC, and 155 PTC on FNAC) and that were confirmed as PTC by matched FFPE sample analysis were evaluated for the BRAF V600E mutation using the LightCycler (Roche Molecular Biochemicals, Mannheim, Germany) PCR method. Nondiagnostic or AUS thyroid nodules on initial FNAC results were consulted for surgery when positive BRAF mutation results were observed or repeated follow-up FNAC results were nondiagnostic, AUS, suspicious PTC, and PTC.

Student’s *t* test, chi-squared test, and multivariate logistic regression analysis were used to assess differences between the nodules with false-negative BRAF V600E mutation results and the others. *P* values < 0.05 were considered statistically significant. All statistical analyses were performed using the SPSS software suite (version 18.0; SPSS Inc., Chicago, IL, USA).

## Results

### 1. BRAF V600E mutation results (Table 2)

Of the 221 PTC nodules, 150 (67.9%) were BRAF V600E mutation positive on FNAC and 185 (83.7%) were BRAF V600E mutation positive on FFPE sample analysis. A total of 37 nodules (16.7%) had discordant results. The sensitivity, specificity, PPV, and NPV for BRAF V600E mutation testing on FNAC were 80.5, 97.2, 99.3, and 49.3%, respectively. A total of 36 nodules (16.3%) were BRAF V600E mutation negative on FNAC but BRAF V600E mutation positive on FFPE sample analysis.

**Table 2:** Comparison of BRAF testing results on FNAC with those on FFPE tissue

		On FFPE tissue	
BRAF status		Positive	Negative
On FNAC	Positive	149 TP	1 FP
	Negative	36 FN	35 TN

Sensitivity = 149/185 = 80.5%

Specificity = 35/36 = 97.2%

Positive predictive value = 149/150 = 99.3%

Negative predictive value = 35/71 = 49.3%

*TP* true-positive, *FP* false-positive, *FN* false-negative, *TN* true-negative

### 2. Comparison of nodules with false-negative BRAF results on FNAC and the other nodules (Student’s *t* test and chi-squared test) (Table 3)

Using the Student’s *t* test and chi-squared test, we performed univariate analysis to find risk factors for false-negative BRAF V600E mutation results on FNAC. The risk factors identified in univariate analysis were age, Bethesda classification, and subtypes of PTC.

**Table 3:** Comparison of cases with false-negative BRAF results and other cases

Variable	Group 1 (*n* = 36)	Group 2 (*n* = 185)	*P* value
	(FN group)	(TP + FP + TN group)	
Gender (F/M)	33/3 (91.7%)	146/39 (78.9%)	0.121
Age	51.7 ± 9.5	45.6 ± 12.8	0.002
Tumor size	0.78 ± 0.72	0.93 ± 0.70	0.241
Extrathyroidal extension	17 (47.2%)	56 (30.3%)	0.074
Lymph node metastasis	20 (55.6%)	92 (49.7%)	0.647
Bethesda system			< 0.001
Nondiagnostic	10 (27.8%)	6 (3.2%)	
Benign	0	0	
AUS	4 (11.1%)	7 (3.8%)	
(Suspicious) FN	0	0	
Suspicious	11 (30.6%)	28 (15.1%)	
Malignant	11 (30.6%)	144 (77.8%)	
Subtype of PTC			0.036
Conventional	30 (83.3%)	131 (70.8%)	
Follicular variant	4 (11.1%)	43 (23.2%)	
Clear cell variant	0	3 (1.6%)	
Oncocytic variant	0	7 (3.8%)	
Tall cell variant	2 (5.6%)	1 (0.5%)	

*FN* false-negative, *TP* true-positive, *FP* false-positive, *TN* true-negative

Patients in the false-negative group (mean age, 51.7 years) were significantly older than the others: true-positive, false-positive, and true-negative groups (mean age, 45.6 years; *p* = 0.002). And, there were more indeterminate FNAC cases (nondiagnostic, AUS, and suspicious PTC) cases in false-negative group (*p* < 0.001). PTC subtypes were also the risk factors for false-negative BRAF V600E mutation results. (*p* = 0.036). However, gender, mean tumor size, presence of extrathyroidal extension, and lymph node metastasis were not significantly different between the groups (Table 3).

### 3. Risk factors associated with false-negative BRAF results (multivariate logistic regression) (Table 4)

Using the multivariate logistic regression method, we analyzed the risk factors for false-negative BRAF V600E mutation results on FNAC. The risk factors in multivariate analysis were age and indeterminate FNAC results (nondiagnostic, AUS, and findings suspicious for PTC). Conventional PTC and tall cell variant PTC subtypes were also the risk factors for false-negative BRAF V600E mutation results on FNAC. The odds ratio with 95% CI and *p* value are listed in Table 4.

**Table 4:** Risk factors associated with false-negative BRAF results

Variable	Odds ratio (95% CI)	*P* value
Age	1.053 (1.012–1.099)	0.012
Bethesda system classification		
Nondiagnostic	32.463 (8.690–140.390)	< 0.001
AUS	10.598 (2.049–54.824)	0.004
Suspicious PTC	3.963 (1.305–12.158)	0.015
PTC	1 (reference)	
Types of PTC		
Conventional	1 (reference)	
Follicular variant	0.218 (0.053–0.712)	0.019
Tall cell variant	33.070 (1.679–952.321)	0.018

## Discussion

The primary goal in evaluating patients with thyroid nodule is the exclusion of thyroid malignancy. Although FNAC is the standard diagnostic method for the differential diagnosis of thyroid malignancy [5–7], it has several limitations. The results of FNAC are indeterminate in up to 30% of cases, and these patients often undergo unnecessary surgery for a benign lesion [5, 8, 9]. Accordingly, the performance of FNAC needs improvement. In clinical settings, especially when FNAC shows suspicious findings, several markers would be helpful adjuncts in discriminating between benign and malignant nodules [18, 19]. An ideal marker is one that can be used in cases of indeterminate thyroid FNAC results to predict a nodule’s benign or malignant state to avoid diagnostic surgery and allow therapeutic surgery to be performed [17–19]. In some studies, molecular tests that “rule-in” for malignancy such as BRAF mutation status, and tests that “rule-out” such as the Gene Expression Classifier helped to facilitate appropriate management [18–20]. However, markers with perfect sensitivity and specificity have not been identified to date [19, 20].

Among several proposed markers, the BRAF V600E mutation is the only one with almost 100% specificity for PTC and is thus a potentially accurate marker for the rending of indeterminate thyroid FNAC to diagnostic cytology [11–14, 17]. The BRAF V600E mutation occurs in 45–80% of sporadic PTC cases but never in either benign lesions such as follicular adenomas, and nodular goiters, or follicular thyroid carcinoma (FTC) [12–14]. Thus, positive BRAF V600E mutation result confirms a diagnosis of PTC.

In the present study, 150 nodules (67.9%) were BRAF V600E mutation positive on FNAC compared to 185 (83.7%) on FFPE sample analysis. This high BRAF V600E mutation positive rate is the distinguishing feature of Korean populations in several previous reports [21–23]. And, the discordance rate was 16.7% (37 cases): 36 nodules (16.1%) were BRAF V600E mutation negative on FNAC but BRAF V600E mutation positive on FFPE and 1 nodule (0.5%) was mutation positive on FNAC but mutation negative on FFPE. When reviewing initial nondiagnostic or AUS lesions with BRAF V600E mutation negative on FNAC, surgery was performed due to the other multifocal PTC nodules, FNAC proven metastatic lymph nodes, and suspicious PTC or PTC results on repeated FNAC.

The risk factors for false-negative BRAF V600E mutation results with FNAC identified in univariate analysis were age, indeterminate FNAC results (nondiagnostic, AUS, and findings suspicious for PTC), and subtypes of PTC (Table 3). The mean age was higher in the false-negative group (51.7 vs. 45.6 years; *p* = 0.002). False-negative results were more common in nondiagnostic cases (27.8 versus 3.2%), AUS cases (11.1 versus 3.8%), and findings suspicious for PTC cases (30.6 versus 15.1%) on FNAC (*p* < 0.001). Moreover, false-negative cases were more often observed in conventional PTC and tall cell variant PTC subtypes (*p* = 0.036). In multivariate analysis, the risk factors identified were age and indeterminate FNAC results (nondiagnostic, AUS, and findings suspicious for PTC). Conventional PTC and tall cell variant PTC subtypes were also the risk factors of false-negative BRAF V600E mutation results (Table 4).

Though positive BRAF V600E mutation result confirms a diagnosis of PTC, wild-type BRAF does not exclude PTC or FTC [12–14, 24, 25]. Moreover in the present study, false-negative results on FNAC were more common in indeterminate FNAC results (nondiagnostic, AUS, and findings suspicious for PTC). The negative BRAF mutation results in indeterminate FNAC have to be even more neglected than expected. Further evaluations such as core biopsy, US feature, other molecular profile, and diagnostic surgery (in cases of repeated nondiagnostic, repeated AUS, and suspicious PTCs) should be considered in these cases [18, 19, 24]. In our center, repeated FNAC or additional core biopsy are applied for initial nondiagnostic or AUS lesions with BRAF mutation negative on FNAC. And, diagnostic surgery with intraoperative frozen biopsy was applied for suspicious PTC lesions with BRAF mutation negative on FNAC.

False-negative results were observed more often in old age with odds ratio 1.053 (CI 1.012–1.099). When interpreting the BRAF mutation results, age should be considered also. False-negative results come out much more in indeterminate FNAC results and come out much less in follicular variant PTC subtype, while much more in conventional and tall cell variant PTC subtypes. Diagnostic or therapeutic surgery can be considered for these cases. Subtypes of PTC are closely related to BRAF mutation rate. As shown in Table 1, the majority of the PTCs in the present study were the conventional type (72.9%) and follicular variant type (21.3%). The conventional PTC type has a relatively high BRAF mutation rate, while the follicular variant type has a relatively low BRAF mutation rate. Therefore, there can be a bias. Also, the number of follicular, clear cell, and other variants are substantially smaller than that of conventional PTC. This can affect their number in a cross-section study. Their BRAF V600E false-negative rate among all conventional PTC is 18%. Further investigations with more large cases are needed to know the clinical implications of these results.

We encountered one false-positive case (53-year-old woman with a 1.2-cm oncocytic variant PTC). This false-positive case may be resulted from a technical error. The LightCycler PCR method has a limitation because a single base pair change in the BRAF mutation was detectable down to the level of 25% of tumor cells when a homozygous mutant cell line was used as control [12, 26, 27]. As such, the detection of the heterozygous BRAF V600E mutation in samples containing < 50% of tumor cells may not be accurate [27]. Also, BRAF V600E test results could be contributed in part by the assay’s technical limitation. The other limitation of our study is that we did not perform BRAF mutation testing in benign and follicular neoplasm cases. Our study is limited by its retrospective design. We cannot perform BRAF mutation testing for benign cases because national health insurance service does not cover. A well-designed prospective study with informed consent of patients will be essential for more informative results.

## Conclusion

BRAF V600E mutation analysis may be a useful adjunct technique for confirming the diagnosis of papillary thyroid carcinoma. However, the false-negative rate of BRAF mutation testing with FNAC for thyroid nodules is increased in cases of old age, indeterminate FNAC pathology results, and certain PTC subtypes. Therapeutic surgery can be considered for these cases. **A** well-designed prospective study with informed consent of patients will be essential for more informative results.
